# Time-Perception Network and Default Mode Network Are Associated with Temporal Prediction in a Periodic Motion Task

**DOI:** 10.3389/fnhum.2016.00268

**Published:** 2016-06-02

**Authors:** Fabiana M. Carvalho, Khallil T. Chaim, Tiago A. Sanchez, Draulio B. de Araujo

**Affiliations:** ^1^Department of Philosophy, University of Sao Paulo (USP)Sao Paulo, Brazil; ^2^Department of Radiology, University of Sao Paulo (USP)Sao Paulo, Brazil; ^3^Department of Radiology, Federal University of Rio de Janeiro (UFRJ)Rio de Janeiro, Brazil; ^4^Brain Institute/Hospital Universitario Onofre Lopes, Federal University of Rio Grande do Norte (UFRN)Natal, Brazil

**Keywords:** temporal expectation, temporal prediction, attention, internal model, fMRI

## Abstract

The updating of prospective internal models is necessary to accurately predict future observations. Uncertainty-driven internal model updating has been studied using a variety of perceptual paradigms, and have revealed engagement of frontal and parietal areas. In a distinct literature, studies on temporal expectations have also characterized a time-perception network, which relies on temporal orienting of attention. However, the updating of prospective internal models is highly dependent on temporal attention, since temporal attention must be reoriented according to the current environmental demands. In this study, we used functional magnetic resonance imaging (fMRI) to evaluate to what extend the continuous manipulation of temporal prediction would recruit update-related areas and the time-perception network areas. We developed an exogenous temporal task that combines rhythm cueing and time-to-contact principles to generate implicit temporal expectation. Two patterns of motion were created: periodic (simple harmonic oscillation) and non-periodic (harmonic oscillation with variable acceleration). We found that non-periodic motion engaged the exogenous temporal orienting network, which includes the ventral premotor and inferior parietal cortices, and the cerebellum, as well as the presupplementary motor area, which has previously been implicated in internal model updating, and the motion-sensitive area MT+. Interestingly, we found a right-hemisphere preponderance suggesting the engagement of explicit timing mechanisms. We also show that the periodic motion condition, when compared to the non-periodic motion, activated a particular subset of the default-mode network (DMN) midline areas, including the left dorsomedial prefrontal cortex (DMPFC), anterior cingulate cortex (ACC), and bilateral posterior cingulate cortex/precuneus (PCC/PC). It suggests that the DMN plays a role in processing contextually expected information and supports recent evidence that the DMN may reflect the validation of prospective internal models and predictive control. Taken together, our findings suggest that continuous manipulation of temporal predictions engages representations of temporal prediction as well as task-independent updating of internal models.

## Introduction

Efficient interaction between an agent and its surrounding relies heavily on the use of prospective internal models to enhance processing of expected stimuli, and on the ability to update these models to achieve the desired outcome (Verschure et al., 2014). Whenever violations of predictions are found (or violations about how we expect the external world to operate), the new perceptual evidence must be integrated into the existing model.

The ability to dynamically adjust an internal model involves: (1) detection of stimulus change; (2) inhibition of the current model; and (3) its updating with the new relevant information (Verbruggen et al., 2010; Hartwigsen et al., 2012). The neural basis of internal model updating has been investigated by several studies. For instance, using a spatial-orienting task O’Reilly et al. (2013) identified the anterior cingulate cortex (ACC) and the adjacent pre-supplementary motor area (pre-SMA) as being specifically associated with uncertainty-driven updating of internal models in a changeable environment. McGuire et al. (2014) reported activity increase in the superior parietal lobule (SPL), anterior prefrontal cortex and cerebellum, related to internal model adjustment, using a predictive-inference task. Actually, the neural basis of internal model updating has been suggested in a Posner task adapted to include temporal attention (Coull et al., 2000). Invalid trials containing delayed targets triggered shifting of attention to a later time point. This attentional shift can be seen as internal model updating since it was caused by changes in contextual rules which make old internal models no longer valid in the novel context (Courville et al., 2006; O’Reilly et al., 2013). Distinct brain areas which responded preferentially to these delayed targets include the premotor cortex, dorsolateral and ventrolateral prefrontal cortices, SMA, SPL and putamen.

Prospective internal models about the timing of events are built up from expectations, and are often performed implicitly. In fact, perceptual implicit timing, or temporal expectations, has been formally split into “endogenous” and “exogenous” modes depending on how temporal expectation is established and manipulated (for a review, see Coull and Nobre, 2008). Endogenous tasks make use of overt, informative pre-cues that signal when an event (target) will occur, which are deliberately used by the subject to direct attention to that point in time. On the other hand, exogenous tasks make use of stimuli with an ongoing and predictable temporal dynamics, which is a covert temporal cue that incidentally generates temporal anticipation. For instance, a pedestrian is able to extrapolate motion trajectories, such as judging future positions of vehicles, to successfully avoid collisions when crossing a busy road. This ability allows precise temporal allocation of attention, which optimizes behavior by improving action preparation and execution (Nobre et al., 2007).

Processes of temporal expectations have been typically investigated using endogenous, symbolically-cued tasks adapted from spatial attention studies. This modular type of paradigm, although well validated, is not representative of a real world experience. More naturalistic paradigms require subjects to use exogenous temporal information to anticipate the exact instant in time an event will occur in order to respond appropriately (Assmus et al., 2005; Coull et al., 2008; O’Reilly et al., 2008; Rohenkohl et al., 2011). The time-perception network engaged by exogenous orienting of attention includes the inferior prefrontal cortex, pre-SMA, dorsal and ventral premotor cortices, inferior parietal lobule (IPL), putamen and cerebellum (for a review, see Coull et al., 2011 and for a meta-analysis, see Wiener et al., 2010).

To our knowledge, no studies on updating of prospective internal models have demonstrated modulation of activity based solely on manipulation of temporal prediction. In the present study, we used functional magnetic resonance imaging (fMRI) to investigate whether the continuous manipulation of temporal expectations would recruit brain regions known to be involved in updating of internal models, and exogenous temporal prediction. We developed an exogenous temporal task that combines rhythm cueing and time-to-contact principles to generate implicit temporal expectation. Time-to-contact tasks consist in judging the instant of collision between objects, and have been employed in studies of spatio-temporal integration (Assmus et al., 2005; Coull et al., 2008). We tested how the estimate of the time-of-arrival (ToA) of a simple pendulum would be affected by unpredictable changes in speed of its swing from one semi-period to the next. By testing the non-periodic against the periodic motion condition we intended to reveal the pattern of brain activity that would represent the effect of non-stop updating of the temporal domain upon the internal model updating and temporal prediction networks.

Despite the prime aim of the present study was to test whether manipulation of temporal prediction would recruit areas involved in internal model updating and exogenous temporal expectation, we also carried out the inverse analysis in which the periodic motion was tested against the non-periodic motion condition. Our purpose with this exploratory analysis was to catch glimpse at the regions which the highly predictive condition (periodic motion) would lead to more activity than the condition where continuous updating of the internal model was required. The difference between the two conditions would reflect the network more engaged in familiar—or predictable—circumstances compared to novel and unpredictable circumstances (Tops et al., 2014). We hypothesize that, just as the non-periodic condition would rely on timing and updating-guided control, the periodic condition would emphasize predictive control guided by the internal models shaped during learning in previous trials.

## Materials and Methods

### Subjects

Sixteen right-handed adult subjects (10 males; mean age 25.4 years ranging from 19 to 32) participated in the fMRI experiment. All had normal or corrected-to-normal vision and were screened for past and present history of neurological, psychiatric or medical problems, as well as current medication use. All volunteers gave their written, informed consent and experimental procedures were approved by and carried out in accordance with the Ethics and Research Committee of the University of Sao Paulo at Ribeirao Preto (No. 10.392/2006).

### Experimental Procedure and Design

**Figure 1:**
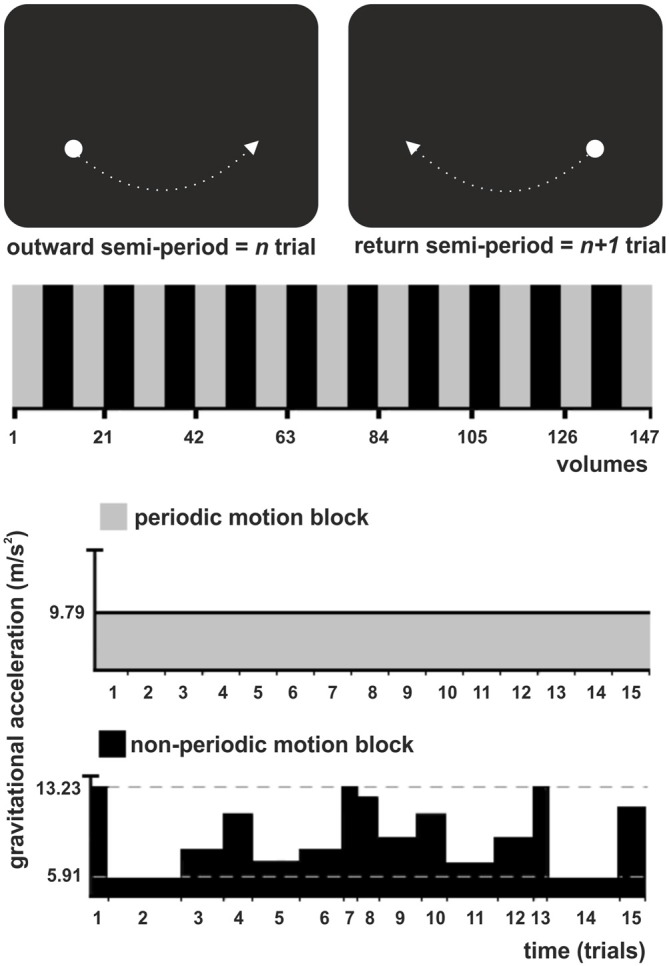
**Experimental task illustration. (Top)** The stimulus was a white circle evolving as a physical pendulum against a black background. A trial corresponded to a semi-period of the pendulum (outward or return), finishing when the pendulum reached the maximum height. Subjects had to intercept the ascending pendulum at the expected time-of-arrival (ToA) at the known spatial location (maximum height) with a button press. **(Middle)** The experiment consisted of one experimental condition (non-periodic motion, black) and the baseline (periodic motion, gray), alternately shifted throughout the task in a standard block design. **(Bottom)** During the baseline, the pendulum exhibited a harmonic oscillation, with a constant gravitational acceleration (GA) and trials of constant length. During the non-periodic blocks, in each trial, the pendulum could take one of eight possible different constant GA values, generating trials with variable lengths.

Stimulus generation and recordings of response times were performed in Presentation software (Neurobehavioral Systems, Inc., San Francisco, CA, USA). The stimulus was a white circle (diameter 1.2°) evolving as a physical pendulum against a black background (Figure 1). Subjects viewed the stimulus on a translucent back-projected screen via a 45° angled mirror fixed onto the head coil. The distance between the screen and the subject’s eyes was 2.5 m.

A trial corresponded to a semi-period of the pendulum (outward or return), finishing when the pendulum reached the maximum height. Subjects were asked to maintain fixation at the center of a screen, where a cross was presented at the beginning of the experiment. They were instructed to press a button as accurate as possible whenever the pendulum reached its maximum height, i.e., to intercept the ascending target at the expected ToA at the known spatial location (right or left side). In all conditions, the pendulum moved with a constant spatial trajectory, which allowed subjects to direct their attention to a specific spatial location. Therefore, spatial expectation was always present and constant throughout the task. Exogenous temporal expectation was manipulated by changing the pendulum acceleration from trial to trial. Subjects had to use implicit (speed)—rather than explicit or cued information—to prospectively direct their attention to the instant the pendulum would reach its maximum height (interception point).

There was no interval between trials, since the pendulum oscillated continuously throughout the task. The experiment consisted of one experimental condition, the non-periodic motion, and an active baseline (periodic motion), alternately shifted throughout the task in a standard block design (Figure 1, Middle). During baseline, the pendulum exhibited a harmonic oscillation, with a constant acceleration caused by a gravitational acceleration (GA) of 9.79 m/s^2^ generating trials with a constant length of 1540 ms. The non-periodic condition was created by manipulating the value of GA. During non-periodic trials, the pendulum took one of eight possible different GA values, ranging from 5.91 to 13.23 m/s^2^, generating trials with variable lengths (ranging from 1340 to 2000 ms; Figure 1, Bottom). Independently of the GA value, target acceleration and deceleration was consistent with natural gravity, i.e., the pendulum always decelerated while moving up and accelerated while moving down. Importantly, the average GA for the non-periodic conditions was 9.79 m/s^2^. The whole experimental task was composed of 21 blocks, each containing 15 trials.

### fMRI Methods

#### Image Acquisition

All images were acquired in a 1.5T (Magneton Vision, Siemens, Erlangen, Germany) whole body MRI system, equipped with a standard TX/RX head coil. Echo-planar imaging (EPI) were acquired in the axial plane, and consisted of 16 slices (voxel size of 3.475 × 3.475 × 6 mm), covering the whole brain, with the following parameters: repetition time (TR) = 3000 ms, echo time (TE) = 60 ms, field of view (FOV) = 220 mm, flip angle = 90°. The whole experiment consisted of 149 volumes acquired while the subject performed the task. Individual T1-weighted, 3D-gradient-echo sequence multiplanar reconstruction (MPR), structural images were acquired for each participant (256 sagittal slices *TR* = 9.7 ms, *TE* = 4 ms, flip angle = 12°, FOV = 256, matrix = 256 × 256 with 1.0 mm^3^ isotropic voxels).

#### fMRI Data Preprocessing

EPI images were preprocessed, analyzed and visualized using BrainVoyager QX 2.8 Software package (Brain Innovation, Maastricht, Netherlands). The first two volumes were discarded to preclude T1 saturation effects. Preprocessing included three dimensional motion correction, linear trend removal and temporal high-pass filtering at 0.01 Hz, and slice-scan-time correction with a sinc interpolation. For multi-subject analysis, a spatial smoothing with a 4 mm full width at half maximum (FWHM) isotropic Gaussian kernel was applied to increase signal-to-noise ratio. Anatomical and functional volumes were normalized to the Talairach and Tournoux (1988) stereotaxic coordinate system. Anatomical images of one subject, selected from the gray-white matter segmentation profile, were used for reconstruction and inflation of the cortical surface from both hemispheres. The resulting inflated representation was then used as reference meshes for projecting functional statistical maps.

### fMRI Data Analysis

#### Whole-Brain Analysis

One single predictor was created for the experimental (non-periodic) condition. The signal time course of each subject was modeled with a boxcar function convolved with a double-gamma hemodynamic response function. Voxel-wise standard analysis was performed using a general linear model (GLM) in order to determine the relative contribution of the non-periodic condition predictor, to the observed voxel time courses. The contrast was specified to test single beta weights—we built up the contrast [non-periodic > periodic] to test in which areas the non-periodic condition would lead to significant greater beta relative to the active baseline (periodic motion). In addition, we evaluated the opposite contrast [non-periodic < periodic] to test in which areas the periodic condition would lead to significant greater betas with respect to the non-periodic condition. For the contrast [non-periodic > periodic], GLM group-level analysis (random effect analysis, one-sample Student’s *t*-test) was performed based on the beta values from individual analysis. *t-*statistic maps were corrected for multiple-comparison at equivalent false discovery rate [q(FDR)] < 0.01]. For the contrast [non-periodic < periodic], GLM group-level analysis (fixed effect analysis, one-sample Student’s *t*-test) was performed. It is important to mention that, for the exploratory “non-periodic < periodic” analysis, we used a simple concatenation approach, which has a higher statistical power, and only represents within-subject variance. Also, it limits the results to the group of subjects studied. We also adopted a more lenient statistical threshold, but still corrected for multiple comparisons (q(FDR) < 0.05).

To investigate further changes between trials, we analyzed each block of the periodic condition as an independent condition. Hence, in this case, the GLM model contained 11 predictors. We then, created three contrasts modeling a pair of blocks at the beginning, middle and end of the experiment, corresponding to three experimental time points (blocks 1 and 2, 5 and 6, 9 and 10, respectively). The three GLM group-level analyses were performed using a fixed effect approach, and the *t-*statistic maps were corrected at q(FDR)< 0.05. This whole-brain analysis was followed by the extraction of the PCC/PC ROI beta weights and *post hoc* pairwise *t*-contrasts (described below).

#### Region of Interest (ROI) Analysis

ROI analysis was performed to investigate: (1) the temporal correlation between a task-positive ROI (middle temporal region: MT+) revealed by the contrast [non-periodic > periodic] and a default-mode network (DMN) ROI [the posterior cingulate cortex/precuneus: PCC/PC] revealed by the contrast [non-periodic < periodic]; and (2) whether activity in the PCC/PC ROI would increase along the periodic blocks.

For the temporal correlation analysis, MT+ and PCC/PC ROIs were created and extracted from whole-brain analysis maps using the BrainVoyager “Talairach coordinate to VOI” plugin. The ROIs were 8-mm-diameter spheres centered at previously published foci (MT+, *x y z* = 45, −69, −2; Fox et al., 2005; PCC/PC, *x y z* = 0, −52, 30; Fransson, 2006), avoiding the problem of circular analysis (or “double dipping”; Kriegeskorte et al., 2009). The mean percent signal change (beta weights) was obtained by using the predictor as the reference. The time-course averaged across all voxels within each ROI was extracted from both ROIs for each subject, and was then averaged across subjects at each volume of acquisition. A Pearson’s correlation coefficient between the MT+ and PCC/PC mean percent signal change averaged across subjects was calculated.

The PCC/PC beta values were tested amongst the three experimental time points. For that, *post hoc* *t*-tests were performed on the following pairwise contrasts between blocks: 1 and 2 < 5 and 6; 5 and 6 < 9 and 10; 1 and 2 < 9 and 10. The data were corrected for serial autocorrelation using an AR(1) model. We reported *t* statistics and corresponding *p* values.

### Behavioral Data Analysis

Response time errors (RTE) were recorded during the fMRI sessions, and were computed as the difference between the recorded button press time, in each trial, and the actual ToA of the pendulum at its maximum height. Thus, positive and negative values reported correspond to over- and underestimation of the pendulum ToA, respectively.

To investigate whether subject’s performance changed over time, we plotted the RTE as a function of trials for both conditions. Each point in the curve represents the mean RTE ± SEM (absolute numbers for positive and negative RTE) for the eleven trials of the given condition. A repeated measures ANOVA (periodicity × trials; Greenhouse-Geisser correction) was calculated to test the main effect of time as well as the interaction between time and condition.

## Results

### Behavioral Results

**Figure 2:**
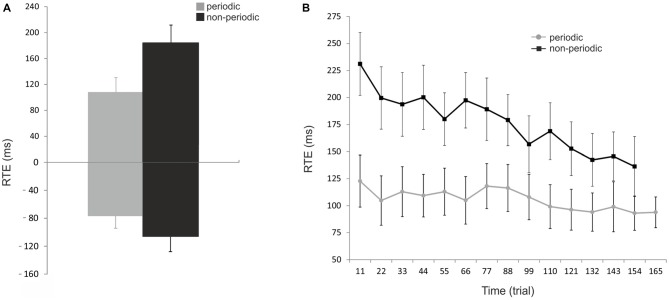
**Behavioral results. (A)** Effect of periodicity of motion on performance. Reaction time error (RTE) represents the accuracy of the subject in estimating the pendulum interception instant; RTEs with positive and negative values correspond to over- and underestimation of the pendulum ToA, respectively. The mean RTE (± SEM) to periodic trials (gray bars) were significantly shorter than that to non-periodic trials (black bars) for both positive and negative values. **(B)** Effect of trial number on performance. Mean RTE (± SEM) as absolute numbers and averaged for every 11 trials across all subjects for both conditions (periodic: gray line; non-periodic: black line). The interception timing becomes significantly more accurate over time in both conditions but with a stronger effect of trial repetition in the non-periodic condition. Periodic: [*F*_(8.44,92.82)_ = 4.35, *p* < 0.005]; Non-periodic: [*F*_(8.77,96.47)_ = 4.54, *p* < 0.0001]; Interaction time * condition: [*F*_(8.21,90.26)_ = 3.15, *p* = 0.003].

Figure 2A shows the RTE averaged across subjects for the periodic and non-periodic trials. Due to a malfunction of the response pad, button presses given by three subjects were only partially recorded and were, therefore, excluded from the analysis. RTE with positive and negative values correspond to over-and under-estimation of the ToA, respectively. Mean RTE of periodic trials (107.5 ± 23.2 ms, −76.3 ± 18.1 ms, mean ± SEM) was significantly shorter than that of non-periodic trials (184.3 ± 27.4 ms, −105.5 ± 22.4 ms, mean ± SEM), for both positive (paired-samples *t*-test; *t*_(145)_ = −13.9, *p* < 0.00001), and negative (paired-samples *t*-test; *t*_(111)_ = −2.9, *p* < 0.005) responses. Moreover, the interception time was underestimated in 5.7% of periodic trials and 9.5% of non-periodic trials indicating that subjects often responded after ToA in both conditions.

Figure 2B shows how subject’s performance changed over time. RTE was significantly dependent on time for both periodic [*F*_(8.44,92.82)_ = 4.35, *p* < 0.005], and non-periodic conditions [*F*_(8.77,96.47)_ = 4.54, *p* < 0.0001]. These results demonstrate that estimation becomes significantly more accurate over time for both conditions. An interaction between time and condition [*F*_(8.21,90.26)_ = 3.15, *p* = 0.003] showed a stronger effect in the non-periodic condition.

### fMRI Results

#### Continuous Manipulation of Temporal Expectations Recruits a Right-Lateralized Timing Network

**Figure 3:**
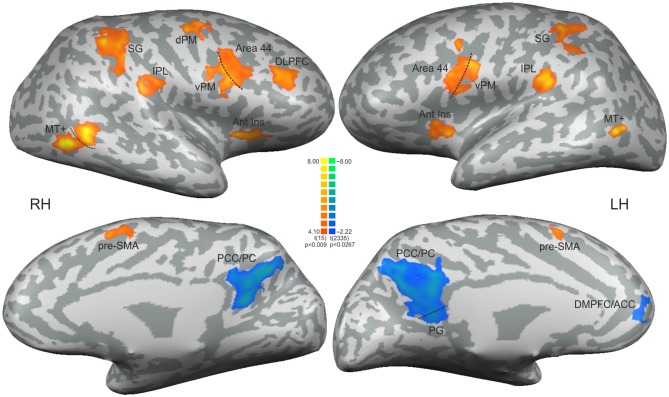
**Statistical significant clusters associated with the contrast [non-periodic > periodic] (orange-yellow scale) and the contrast [non-periodic < periodic] (blue-green scale).** All clusters are listed in Table 1. The statistical maps are projected onto a 3D surface rendering of the standard normalized brain of one subject. DLPFC, dorsolateral prefrontal cortex; Ant Ins, anterior insula; vPM, ventral premotor area; dPM, dorsal premotor area; IPL, inferior parietal lobule; SG, supramarginal gyrus; MT+, middle temporal region; pre-SMA, presupplementary motor area; PCC/PC, posterior cingulate cortex/precuneus; PG, parahippocampal gyrus; DMPFC/ACC, dorsomedial prefrontal cortex/anterior cingulate cortex. RH, right hemisphere; LH, left hemisphere.

Updating the ToA judgment in the non-periodic motion (given by the comparison with the active baseline) recruited regions in the frontal, parietal and temporal lobes, bilaterally, but more extensive in the right hemisphere. Exclusive right hemisphere activity was observed in the dorsolateral prefrontal cortex (DLPFC—BA 9/46) and in the dorsal premotor cortex (dPM—BA 6/9). Bilateral frontal activity included the ventral premotor cortex (vPM—BA 6), the adjacency of area BA 44 (middle frontal gyrus), the presupplementary motor area (pre-SMA—BA 6) and the anterior insula (BA 13). Bilateral activity was also observed in the IPL, and in the supramarginal gyrus (both BA 40). Temporal cortex activity was observed in the middle temporal gyrus mainly over the MT+ region (BA 37; Table 1, Figure 3—regions depicted using an orange–yellow color-scale).

**Table 1:** **fMRI significant clusters, with the respective max *t*-values and peak location (Talairach coordinate), and Brodmann areas (BA) for the contrasts (A) non-periodic > periodic motion and (B) non-periodic < non-periodic motion**.

Anatomical region (Functional region)	Brodmann area	*t-*value	Peak location (*x y z*)
**A. Contrast [non-periodic > periodic]**		
R inferior + middle frontal gyrus (Area 44)	44	5.28	50 15 15
L inferior + middle frontal gyrus (Area 44)	44	5.07	−52 10 21
R middle frontal + precentral gyri (vPM)	6	5.24	44 4 25
L middle frontal + precentral gyri (vPM)	6	5.29	−48 −1 26
R middle frontal gyrus (DLPFC)	9/46	5.29	35 32 30
R superior frontal gyrus (dPM)	6/9	5.13	30 2 52
R medial frontal gyrus (pre-SMA)	6	5.22	6 4 49
L medial frontal gyrus (pre-SMA)	6	5.01	−5 4 50
R anterior insula	13	6.05	36 16 8
L anterior insula	13	5.36	−33 14 10
R inferior parietal lobule	40	5.41	42 −39 40
L inferior parietal lobule	40	5.22	−49 −43 36
R supramarginal gyrus	40	5.00	58 −29 24
L supramarginal gyrus	40	5.77	−56 −31 30
R middle temporal gyrus (MT+)	37	7.43	50 −61 1
L middle temporal gyrus (MT+)	37	5.64	−45 −67 2
**B. Contrast [non-periodic < periodic]**		
R posterior cingulate gyrus	23/30/31	−3.39	4 −48 21
L posterior cingulate gyrus	23/30/31	−3.84	−4 −52 25
R precuneus	7/31	−3.78	4 −60 29
L precuneus	7/31	−3.71	−4 −63 31
L parahippocampal gyrus	30	−2.92	−10 −44 3
L medial frontal gyrus	9/10	−2.53	−3 52 13
L anterior cingulate gyrus	10/32	−2.46	−3 45 7

Threshold was set at q[FDR] < 0.01 and 0.05 for the two contrasts, respectively. Brain areas are illustrated in Figure 3. vPM, ventral premotor cortex; dPM, dorsal premotor cortex; DLPFC, dorsolateral prefrontal cortex; pre-SMA, presupplementary motor area.

**Figure 4:**
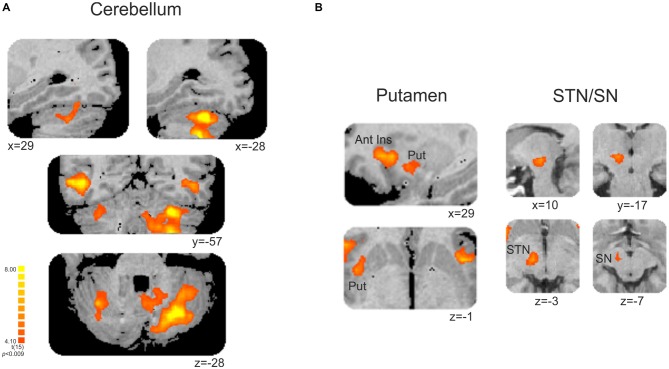
**(A)** Cerebellar and **(B)** subcortical clusters associated with the contrast [non-periodic > periodic]. All significant areas are listed in Table 2. Put, putamen; Ant Ins, anterior insula; STN, subthalamic nucleus; SN, substantia nigra. Right is left.

This contrast (non-periodic > periodic) also showed significant greater activity in the basal ganglia and in the cerebellum. Cerebellar activity was observed bilaterally, but more extensively in the left hemisphere. On the left side, one cluster extended from anterior to posterior cerebellar hemisphere along lobules IV–V, VI and VII B, and also towards the cerebellar tonsil until the vermis (lobule X). The right cerebellar hemisphere showed activity in the culmen and declive (lobules IV–V and VI, respectively; Table 2, Figure 4A). Cerebellar anatomical regions were reported according to Talairach and Tournoux (1988) convention. Right basal ganglia were also recruited, and included the subtalamic nucleus (STN) and the substantia nigra (SN), located in one single cluster, and the putamen (extending to the claustrum; Table 2, Figure 4B).

**Table 2:** **Cerebellar and subcortical fMRI significant clusters, with the respective max *t*-values and peak location (Talairach coordinate), and BA for the contrast non-periodic > periodic motion**.

	Contrast [non-periodic > periodic]	
Anatomical region	*t*-value	Peak location (*x y z*)
**Cerebellum (lobule)**		
R Hemispheric culmen (IV–V)	5.02	35 −49 −28
L Hemispheric culmen (IV–V)	6.82	−33 −59 −26
R Hemispheric declive (VI)	5.09	34 −62 −2
L Hemispheric declive (VI)	6.49	−30 −60 −21
L Inferior semilunar (VII B)	5.14	−24 −62 −39
L Vermal nodule (X)	4.65	−10 −50 −29
L Cerebellar tonsil	5.70	−29 −57 −40
**Basal ganglia**		
R Putamen	5.23	28 2 −1
R Substantia nigra	5.26	9 −15 −7
R Subtalamic nucleus	5.28	10 −17 −3

*Threshold was set at q[FDR] < 0.01. Brain areas are illustrated in Figure 4*.

#### Fulfillment of Temporal Expectations Activates a Left-Lateralized Default-Mode Network

The exploratory contrast [non-periodic < periodic] yielded significant greater activity in medial regions of the Default Mode Network (DMN, Table 1, Figure 3—regions depicted using a blue–green color-scale). These clusters were predominant in the left-hemisphere. Only the PCC/PC was bilateral, although the left PCC/PC cluster was more extensive and included parts of the parahippocampal cortex. Significant greater activity was also found in left hemisphere anterior DMN areas—the dorsomedial prefrontal, cortex (DMPFC) and the ACC. Also, the statistical map using a random effect model for this exploratory analysis is shown in Supplementary Figure 1.

#### PCC/PC: Temporal Anticorrelation with MT+, and Increased Activity Across the Periodic Task

**Figure 5:**
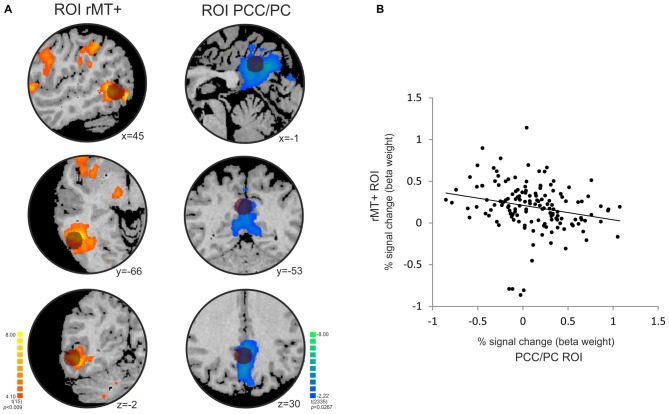
**Temporal anticorrelation between PCC/PC and right middle temporal area (rMT+). (A)** The location of the PCC/PC ROI (x y z = 0, −52, 30; Fransson, 2006) and rMT + ROI (x y z = 45, −69, −2; Fox et al., 2005) used in the analysis is depicted by the dark circles on top of the statistical maps for the contrasts [non-periodic > periodic] (orange-yellow scale) and [non-periodic < periodic] (blue-green scale). **(B)** Correlation plot between the percent signal change extracted from the rMT+ (*y*-axis) and PCC/PC (*x*-axis) ROIs averaged across subjects at each volume of acquisition (*r* = −0.228; *p* = 0.006).

The temporal correlation analysis between the right MT+ and PCC/PC showed a significant negative correlation (*r* = −0.23, *p* = 0.0055; Figure 5). MT+ and PCC/PC were chosen from the non-periodic and the periodic motion active regions, respectively, due to their highest *t-*statistic values (Table 1).

**Figure 6:**
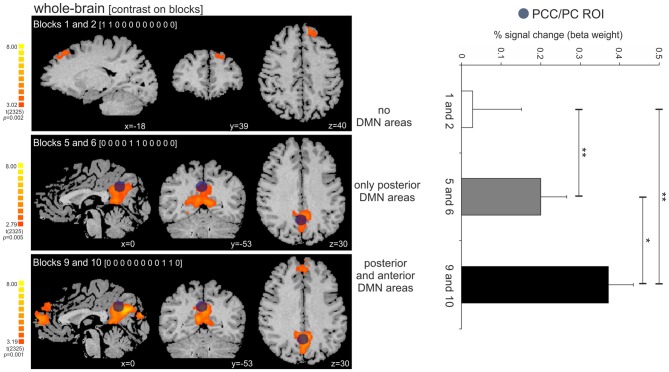
**Activity increase in the PCC/PC ROI across the three tested time-points (beginning, middle and end of the periodic task). (Left panel)** Dark blue circle on top of the statistical maps depicts the location of the PCC/PC ROI used for the three contrasts. The contrasts on the periodic motion blocks revealed clusters in the superior frontal cortex (BA 8; blocks 1 and 2), PCC/PC (blocks 5 and 6; blocks 9 and 10), and DMPFC/ACC (blocks 9 and 10). **(Right panel)** The activity in the PCC/PC ROI significantly increased across the three blocks. *p = 0.01; **p < 0.005.

The activity in the PCC/PC ROI significantly increased across the three tested time-points, corresponding to the beginning, middle and the end of the periodic task (*post hoc* pairwise *t*-contrasts: time-point 1 < 2, *t* = 2.87, *p* = 0.004; time-point 2 < 3, *t* = 2.41, *p* = 0.01; time-point 1 < 3, *t* = 2.96, *p* = 0.003—all tests were corrected for serial autocorrelations using an AR(1) model; Figure 6). At the first time-point, we observed a single significant cluster at BA 8 (peak location: *x* = −18; *y* = 39; *z* = 40). At the second and third time-points, we observed posterior only and posterior/anterior DMN midline areas, respectively. We also correlated the PCC/PC ROI percent signal change with the performance (mean RTE) throughout the periodic motion task. The results showed a significant negative correlation between beta values in the PCC/PC ROI with the periodic motion RTE (*r* = −0.198; *p* = 0.016). These findings suggest that greater PCC/PC activity is associated with more accurate responses.

## Discussion

In the present study, we used a white circle exhibiting pendular motion that could assume two possible patterns: periodic motion (isochronous rhythm), and an irregular, non-periodic motion. Using implicit temporal information (speed), subjects had to intercept the ascending pendulum at the expected ToA at the maximum height of the trajectory. fMRI and behavioral data was inspected in order to investigate whether the continuous manipulation of temporal expectations would recruit brain regions known to be involved in updating of internal models and exogenous temporal expectation. The non-periodic motion tested against the periodic (active) baseline elicited activity mainly in the ventral premotor, inferior parietal and middle temporal cortices, in the pre-SMA, as well as cerebellum and basal ganglia. When periodic motion was tested against non-periodic motion, we observed significant clusters in a subset of midline DMN areas, including the DMPFC and ACC, and more extensively in the PCC/PC.

### Continuous Manipulation of Temporal Expectations—the Right-Hemisphere Preponderance and the Role of Cerebellum

Significant changes were observed across premotor areas, dorsolateral prefrontal cortex, IPL, as well as subcortical and cerebellar regions, when subjects had to continually update their internal models about temporal expectations (non-periodic > periodic). These results suggest that the time-perception network described in previous research on temporal orienting of attention (Coull et al., 2008; O’Reilly et al., 2008; Billington et al., 2011; see Wiener et al., 2010); for a meta-analysis and see Coull et al., 2011; for a review was recruited by the continuous manipulation of temporal expectation. We also found greater bilateral activity in the SPL and pre-SMA, regions which have been reported to participate in uncertainty-driven internal model updating (Coull et al., 2000; O’Reilly et al., 2013; McGuire et al., 2014).

Actually, the pre-SMA (and SMA), in addition to internal model updating, has also been largely implicated in virtually all forms of timing, being recruited in sub- and supra-second interval tasks, both in motor and perceptual paradigms (Wiener et al., 2010). Indeed, the pre-SMA has become a brain area of great interest, particularly for perceptual-action control (Ridderinkhof, 2014). It has been argued that pre-SMA may play an integrative role between attentional/cognitive control and motor systems, since it mediates both attention and motor inhibition required for action updating (Zandbelt et al., 2013; Roberts and Husain, 2015).

Although we observed changes in the activity of the pre-SMA, we did not observe greater ACC activity, related to internal model updating, as reported by O’Reilly et al. (2013). There are at least two important differences in the paradigm of these studies. The first regards the observation that “uncertainty” is not comprised of a single dimension as recent evidence shows different neural correlates involved in representing and manipulating different types of uncertainty (Yu and Dayan, 2005). O’Reilly et al. (2013) situate their updating findings within the estimation uncertainty scope, which is not inherent to the environment and can be reduced by the subject. In the present study, uncertainty was generated by volatility, which is a form of expected uncertainty generated by frequent changes in the environment, causing the subject to learn that changes will occur rapidly, leading to constant updating of “stimulus-response-outcome” rules (Bland and Schaefer, 2012). In our task, the constant updating of “stimulus-response-outcome” was driven by changes in speed, which impelled subjects to adjust the response time to achieve a successful outcome. The ACC has been shown to be engaged in tasks demanding conflict monitoring, or uncertainty estimation where top-down expectations are strongly violated. In such contexts, internal models are not only slightly adjusted, but should be overridden, which could be seen as a mode of extreme updating (Karlsson et al., 2012; Tervo et al., 2014). The second difference is the fact that the change in expectations in the present study was restricted to the temporal domain. This might have preferentially recruited areas which are part of the time perception network, in which pre-SMA is a key component (Wiener et al., 2010; Coull et al., 2011).

Despite the fact that activity was observed in both hemispheres, a clear right preponderance is reported in present study. Usually, temporal expectation, in both endogenous and exogenous tasks, has been associated with a particular left-lateralized set of regions, most consistently left inferior parietal cortex, but also left ventral premotor cortex and cerebellum (Coull and Nobre, 1998; Wiener et al., 2010; Coull et al., 2011). However, it is important to note that fMRI studies have also reported a *right*-lateralized/preponderant prefrontal-parietal network for perceptual prediction in time-to-contact (Billington et al., 2011) and spatiotemporal trajectory tasks (O’Reilly et al., 2008; Beudel et al., 2009).

Explicit timing has been particularly associated with right prefrontal and parietal areas, which demand an overt estimate of stimulus duration, and also the phenomenon of “hazard function”. By definition, the hazard function is the increasing conditional probability that an event will occur given that it has not yet occurred (Nobre et al., 2007). The hazard function concept could be applied to understanding the right hemisphere dominance found in the present study. By knowing beforehand that a new update will be demanded by the upcoming trial—the expectation that the change in speed will certainly occur soon—might have increased the sense of subjective temporal expectation. Given the continuous nature of the task (trials were not temporally separated), it could be argued that, in addition to the temporal expectation used to make a judgment about the ToA within the current trial, subjects could also have built up a parallel expectation towards the start of the next trial. The temporal attentional resources could have been divided: the subjects had to sustain their attention in the current trial to perform the exogenous task while projecting their attention towards the beginning of the next trial, in order to predict the stimulus onset, probably for readiness. In other words, continuous manipulation of temporal expectations present in our exogenous task might have caused attention to be directed to both incidental time (speed for a ToA judgment), associated with left prefrontal-parietal activity, and to the passage of time itself, related to right-lateralized activity.

Importantly, our hypothesis that the right-hemisphere preponderance could have been due to engagement of explicit timing mechanisms implies that the non-periodic motion requested greater endogenous control of attention. Taking into account the evidence of a close causal relationship between attentional control, performance improvement and monitoring (Petersen and Posner, 2012), we can assume sustained attentional engagement by the subjects throughout the task in both conditions (given the performance improvement showed by the behavioral data). Nevertheless, the continuous manipulation of the temporal expectation during the non-periodic motion condition may have created a context of increased attentional demands. However, sustained attention has been described as closely related to continuous update of internal models (Reynolds et al., 2009). Also, for supra-second durations sustained attention is fundamentally necessary and cannot be disentangled from the processing of timing (Coull, 2014). In fact, a recent meta-analysis on sustained attention has provided a putative core network comprising several areas overlapping the time-perception network: SMA, the IPL, cerebellum, putamen and midbrain (Langner and Eickhoff, 2013). Therefore, controlling for high-level cognitive confounds such as sustained attention in second-range timing can be rather challenging. Some studies have tried to control for them by dissociating drug effect on supra-second timing from collateral effects on attentional and mnemonic mechanisms (Coull, 2014). In the present study, the very effect we wanted to test is highly dependent on mechanisms of sustained attention, and we did not further explore a possible confounding effect of attentional control.

We also observed a left-lateralized cerebellar activity, which has been reported in previous studies of exogenous temporal expectations (O’Reilly et al., 2008; Beudel et al., 2009) and internal model update (McGuire et al., 2014). The cerebellum is known to be involved in a number of processes, including motor, sensory as well as cognitive functions (Strick et al., 2009). Purkinje cells are the principal processing unit of the cerebellar cortex, and they play an integrative role in processing information from different deep cerebellar nuclei, which in its turn receive inputs from different cortical brain regions (Apps and Garwicz, 2005). This structural invariance suggests uniformity in information processing, regardless of the specific cortical afferent. It has been proposed that prediction in the cerebellum is not limited to sensory, motor or cognitive processing, but it is rather performed under a general rule given the identical processing mechanism performed by the cerebellum on different neuronal input (Ramnani, 2006; Koziol et al., 2012). Several reviews present evidence that the cerebellum shows strong interconnections with premotor as well as other prefrontal and parietal cortices (Ramnani, 2006; Strick et al., 2009). Therefore, as the cerebellum receives rich contextual information from several cortical areas, it can generate and update predictions taking into account specific and actual spatio-temporal information. Moreover, the connections between cerebellum and cerebrum are crossed between hemispheres, so it is expected to observe left-lateralized cerebellar activity given the right-lateralized brain activity reported.

In addition to the corticocerebellar interaction necessary for the temporal tune of the prospective internal model, the cortico-striato-cortical loop constantly evaluates temporal structures and extracts regularities (Coull et al., 2011). By doing so, the corticostriatal network establishes internal representations that will be used by the cerebellum to generate predictions and update the internal model if necessary. In the present study, we also observed right-lateralized activity in the putamen, SN and STN. The SN and putamen are both components of the nigrostriatal dopaminergic pathway whose involvement in interval timing has been largely described, and the cortico–striatal–thalamic pathway integrates the STN into the timing network (Buhusi and Meck, 2005; Coull et al., 2011). Anatomical evidence shows that the cerebellum has connections with the putamen, which in turn has connections with the pre-SMA (Lehéricy et al., 2004; Hoshi et al., 2005). The pre-SMA integrates the temporal information provided by the cerebellum, and projects to the DLPFC where internal representations and actual temporal information are integrated (re-analysis when incongruent data is perceived), allowing reorganization of perceptual-motor competences (Pochon et al., 2001). Indeed, this operational mode of the corticocerebellar and the corticostriatal networks in evaluating temporal internal models has been suggested to subserve high-level processes that demand fine-tuned temporal prediction, and updating, such as language (Kotz and Schwartze, 2010), motor imagery, and action observation (Gatti et al., 2013).

### High Predictability and the Default-Mode Network

The exploratory analysis (non-periodic < periodic) has shown relative greater of activity in a particular subset of midline DMN areas, including the left DMPFC, the ACC and a more extensive bilateral activity in the PCC/PC. Although the DMN can be reliably identified in resting state data or task-related decreased activity, task-induced increases in DMN regions are observed across internal and external cognitive tasks (Spreng et al., 2009). External information processing has been associated with increased DMN activity in paradigms involving social cognition and also in tasks that demand an active environment monitoring, suggesting that the DMN plays a role in processing contextually expected information (Bar, 2009; Meyer et al., 2012; Tylén et al., 2015). For instance, the same medial regions that are part of the DMN also support the processing of contextual associations during recognition of visual scenes and objects, and form the so-called contextual association network (Bar, 2009). Also, it has recently been shown that the DMN is associated with the comprehension of coherent narratives, playing a central role in integrating fragmented information into larger-scale mental models, while incoherent stories engages the frontoparietal network (Tylén et al., 2015).

Our behavioral and fMRI results indicate that increasing stimulus familiarity results in greater activity of DMN areas. Behaviorally, response accuracy significantly increased as the subjects learned how to predict the regular pendular motion, and the improvement in performance was associated with greater in activity in the PCC/PC region. This result suggests that the more fluent and habitual the prospective internal models get, the less necessity of continuous feedback controlling each behavioral step, thus reducing the degree of attentional engagement to external cues, but still demanding an active monitoring of the environment. In fact, it has been proposed that the posterior medial subsystem composed by the PCC/PC monitors behavior in familiar environments, guided by predictive internal models. Predictive behavior in the PCC/PC allows for increasingly automated action control, as a novel task becomes more predictable after learning, that is, after the formation of reliable internal models (Tops et al., 2014).

The predictability created by the periodic condition could also be approached in terms of task demand. Manipulations of task difficulty within factors as target discriminability, stimulus presentation rate and working memory load have revealed that DMN deactivation magnitude increased with task difficulty (McKiernan et al., 2003), and that the functional connectivity of the PCC to the DMN also changes as a function of task demand (Leech et al., 2011). Task attentional demand is not an alternative interpretation of the DMN activity elicited by the periodic task, but it complements our understanding of the updating of internal models and predictive control in highly expected contexts. Task familiarity is amongst the factors that can influence task processing demands, and in the present study familiarity was achieved through a shift in processing from reactive to predictive perception-action control. The activity in the PCC/PC increased throughout the periodic task (blocks), and the participation of the DMPFC and ACC was only observed in the two last blocks. These findings suggest that subjects gradually engage less mental effort to the task as they learn the stimulus regularity, and the gradual change from reactive to predictive control seems to be a process that selectively recruits areas of the DMN as it moves from performance learning to a partly automated action control.

In addition to the activity of the DMN midline areas, we also found a negative correlation between the PCC/PC and the motion-sensitive area, MT+. The MT+ has been implicated in speed perception (Liu and Newsome, 2005) as well as in temporal attention guided by predictability of stimuli (Fischer et al., 2013), two temporal aspects modulated by the present task. This result is in line with previous reports of an negative correlation between MT+ and the DMN in paradigms which the baseline condition corresponds to the absence of any over task or behavior (Fox et al., 2005; Uddin et al., 2009; however, see Saad et al., 2012 for a controversy about the orthogonality between these two networks in resting-state data). Here we show that such negative correlation is also present when contrasting predictable and unpredictable information processing.

## Conclusion and Limitations

During the task, subjects had to handle continuous manipulation of temporal expectations as a consequence of observing the non-periodic motion of a pendulum, in order to predict the pendulum ToA. The task did not require an explicit temporal estimate but, rather, it elicited mechanisms of exogenous temporal expectation given by the speed of the pendulum. We observed that the continuous manipulation of temporal predictions recruited brain areas known to be involved in updating of internal models, and exogenous temporal expectation (pre-SMA, ventral premotor and inferior parietal cortices, and the cerebellum). Interestingly, we found a right-hemisphere preponderance, which could be understood as the built-up of a parallel expectation towards the start of the next trial (attention to the passage of time itself). However, it also points out to a possible confounding effect of attentional control that has not been further explored in the present study. Taken together, our findings suggest that continuous manipulation of temporal predictions engages representations of temporal prediction as well as task-independent updating of internal models. Moreover, we demonstrated that the periodic motion condition (when compared to the non-periodic motion) yielded activity in the DMN midline areas that increased throughout the task, suggesting that these areas can be modulated by stimulus familiarity.

The lack of a baseline common to both conditions, such as a resting or passive viewing condition is an important limitation of our study. Without a common baseline, conclusions can only be drawn upon a relative contribution between conditions, and does not allow interpreting the results with respect to increased or decreased activity in one condition. The exploratory analysis of the periodic against the non-periodic motion condition would not change the pattern of activity elicited by the manipulation of temporal expectations. Importantly, the limitation caused by the lack of a common baseline and the exploratory analysis conducted using the contrast “non-periodic < periodic” do not detract from our inferences from the current data.

## Author Contributions

Conceived and designed the experiments: FMC, DBdeA. Performed the experiments: FMC. Analyzed the data: FMC, TAS. Contributed with analysis tools: KTC. Wrote the article: FMC, TAS, DBdeA.

## Conflict of Interest Statement

The authors declare that the research was conducted in the absence of any commercial or financial relationships that could be construed as a potential conflict of interest.

## Supplementary Material

Supplementary Figure 1 **Statistical significant clusters associated with the contrast [non-periodic < periodic] using a random-effect model (blue-green scale).** The statistical maps are projected onto a 3D surface rendering of the standard normalized brain of one subject (Top) and onto the normalized anatomical volume (Bottom). The posterior cluster corresponds to the posterior cingulate cortex/precuneus region (PCC/PC). The anterior, smaller clusters correspond to the dorsomedial prefrontal cortex/anterior cingulate cortex (DMPFC/ACC) region. RH, right hemisphere; LH, left hemisphere; A, anterior; P, posterior.
