# Supplementary figures and images for: Time-Perception Network and Default Mode Network Are Associated with Temporal Prediction in a Periodic Motion Task
Source: Front Hum Neurosci. 2016 Jun 2;10:268. doi: 10.3389/fnhum.2016.00268 (PMC4889611; doi:10.3389/fnhum.2016.00268)

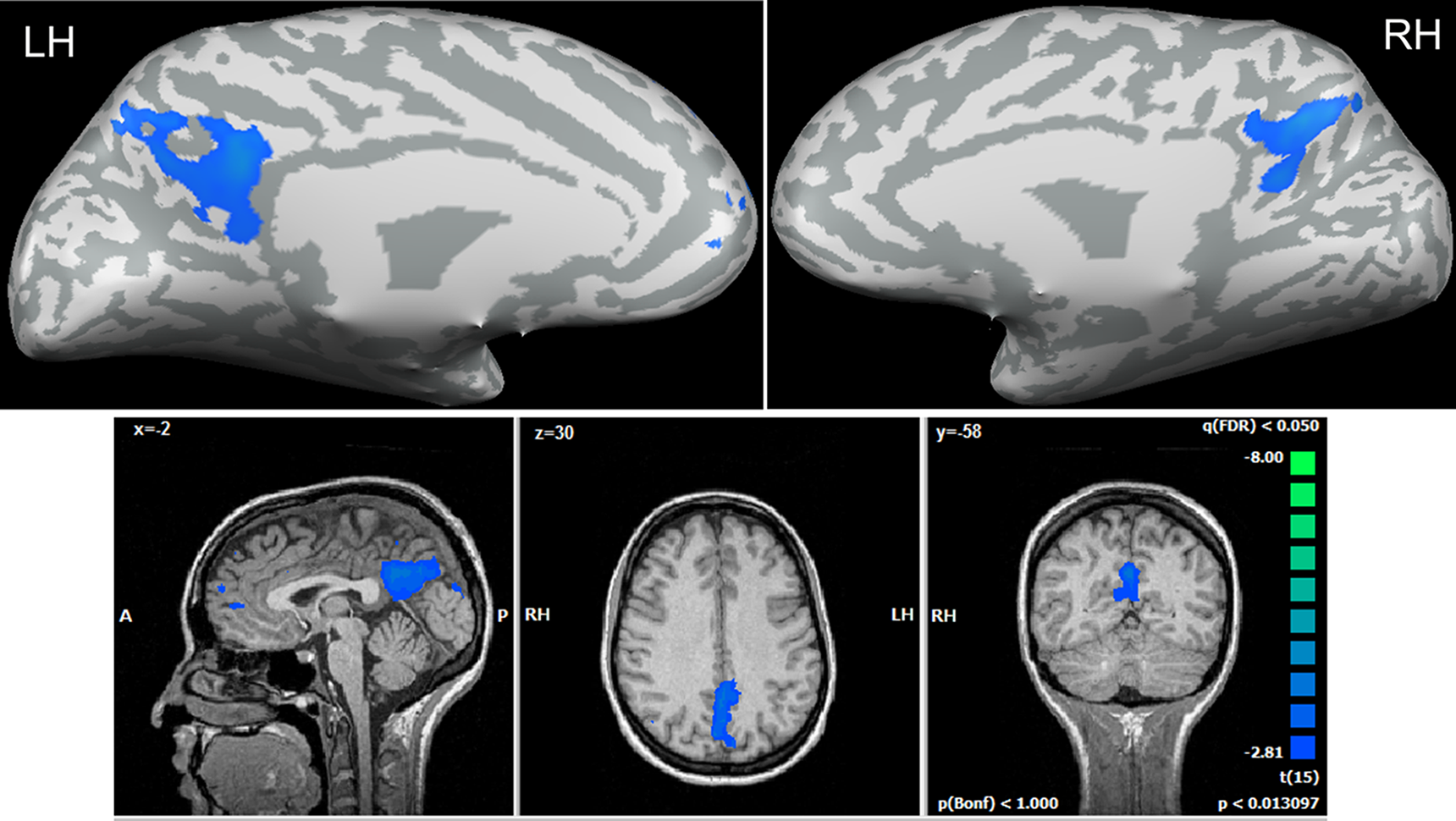

Supplement: Supplementary Figure 1 — Statistical significant clusters associated with the contrast [non-periodic < periodic] using a random-effect model (blue-green scale). The statistical maps are projected onto a 3D surface rendering of the standard normalized brain of one subject (Top) and onto the normalized anatomical volume (Bottom). The posterior cluster corresponds to the posterior cingulate cortex/precuneus region (PCC/PC). The anterior, smaller clusters correspond to the dorsomedial prefrontal cortex/anterior cingulate cortex (DMPFC/ACC) region. RH, right hemisphere; LH, left hemisphere; A, anterior; P, posterior. [file Image_1.tif]
